# The Influence of Robot Verbal Support on Human Team Members: Encouraging Outgroup Contributions and Suppressing Ingroup Supportive Behavior

**DOI:** 10.3389/fpsyg.2020.590181

**Published:** 2020-12-22

**Authors:** Sarah Sebo, Ling Liang Dong, Nicholas Chang, Michal Lewkowicz, Michael Schutzman, Brian Scassellati

**Affiliations:** ^1^Department of Computer Science, University of Chicago, Chicago, IL, United States; ^2^Department of Computer Science, Yale University, New Haven, CT, United States

**Keywords:** psychological safety, inclusion, backchannels, groups and teams, human-robot interaction

## Abstract

As teams of people increasingly incorporate robot members, it is essential to consider how a robot's actions may influence the team's social dynamics and interactions. In this work, we investigated the effects of verbal support from a robot (e.g., “*good idea Salim*,” “*yeah*”) on human team members' interactions related to psychological safety and inclusion. We conducted a between-subjects experiment (*N* = 39 groups, 117 participants) where the robot team member either (A) gave verbal support or (B) did not give verbal support to the human team members of a human-robot team comprised of 2 human ingroup members, 1 human outgroup member, and 1 robot. We found that targeted support from the robot (e.g., “*good idea George*”) had a positive effect on outgroup members, who increased their verbal participation after receiving targeted support from the robot. When comparing groups that did and did not have verbal support from the robot, we found that outgroup members received fewer verbal backchannels from ingroup members if their group had robot verbal support. These results suggest that verbal support from a robot may have some direct benefits to outgroup members but may also reduce the obligation ingroup members feel to support the verbal contributions of outgroup members.

## 1. Introduction

Over the past several decades, researchers have consistently demonstrated that a team's social dynamics are powerful predictors of both the satisfaction of team members and the team's overall performance (Jones and George, 1998; Edmondson, 1999; Woolley et al., 2010; Shore et al., 2011). For example, teams that have an inclusive environment, that value the input from members with diverse backgrounds and experiences, have more committed team members and better performance outcomes (Cho and Mor Barak, 2008; Shore et al., 2011; Sabharwal, 2014). Additionally, teams with high psychological safety (a “shared belief held by members of a team that the team is safe for interpersonal risk taking”) are more successful because team members ask for help, seek feedback, and openly discuss errors (Edmondson, 1999).

As robots increasingly join human teams and collaborate with people on a variety of tasks, it seems reasonable to program these robots with the ability to positively contribute to important team social dynamics, like inclusion and psychological safety in order to maximize team performance. In line with this idea, recent work has discovered robot behaviors that can positively shape specific social dynamics in groups and teams of people, including cohesion (Short and Matarić, 2017), conflict resolution (Shen et al., 2018), conversation dynamics (Traeger et al., 2020), and verbal participation (Tennent et al., 2019). While this body of work has made some important first steps in understanding how robots can influence social dynamics in human-robot teams, little work has investigated how robots might be able to promote greater psychological safety and inclusion among the human members of a human-robot team.

**Figure 1:**
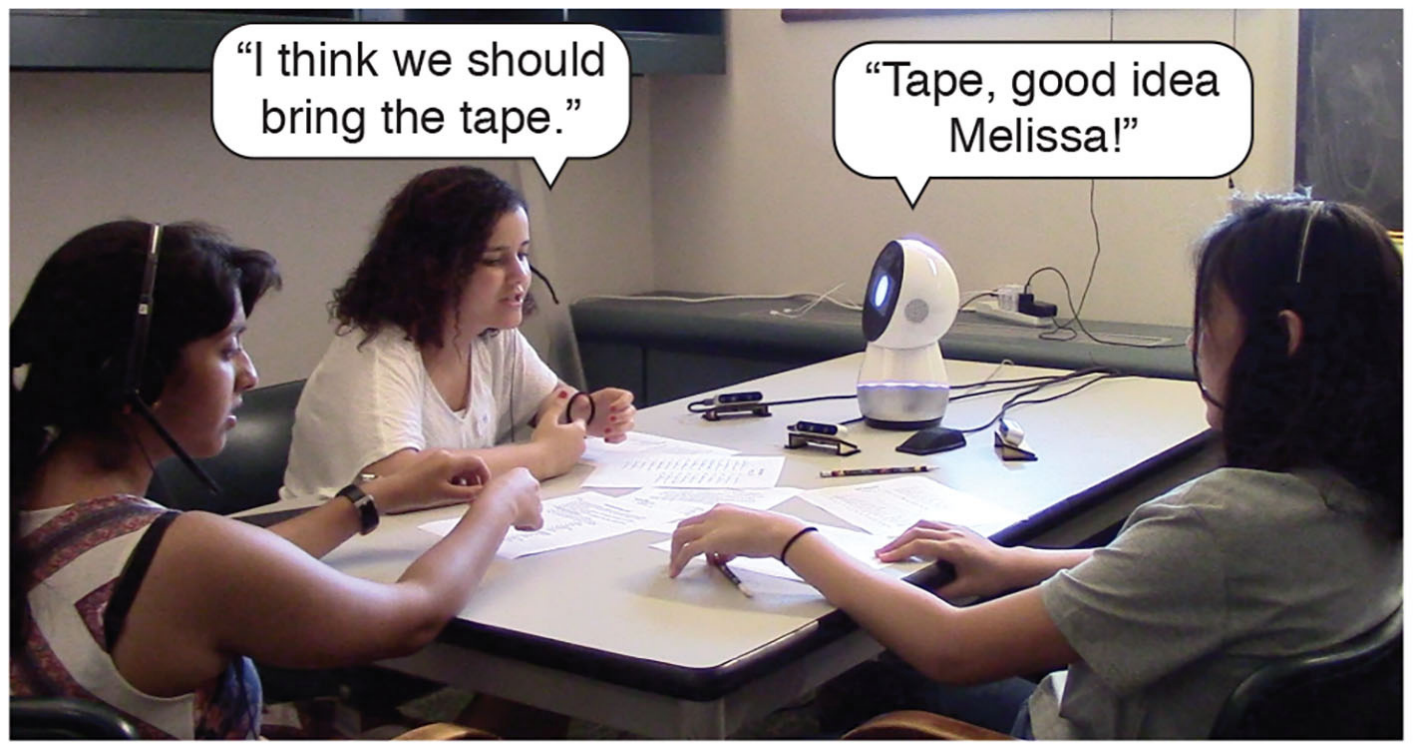
Robot verbal support (e.g., “*Tape, good idea Melissa*”) delivered to human members of collaborative teams resulted in greater verbal engagement from the outgroup member as well as inhibited verbal support from ingroup members to the outgroup member.

In this paper, we present a human subjects study designed to test the effectiveness of robot verbal support (e.g., “*good idea Salim*,” “*yeah*”) on human team members' inclusion and psychological safety in the context of a collaborative task, see Figure 1. We constructed groups that consisted of 2 human ingroup members, 1 human outgroup member, and 1 robot, in order to specifically test whether these robot interventions were helpful to the human outgroup member. We employed a between-subjects experimental design, where some groups interacted with a robot that gave them both task information as well as verbal support and other groups interacted with a robot that only gave them task information. Our results surprised us: we found that while targeted support from the robot encouraged participation from outgroup members, the verbal support from the robot seemed to *suppress* the verbal support the ingroup members would normally give the outgroup member. Our findings provide evidence that robot behavior designed to express verbal support may encourage the participation of outgroup members and also reduce the burden ingroup members feel to support the contributions of outgroup members^1^.

## 2. Background

In this section, we review work examining the dynamics and interactions within human teams and the growing body of work describing robot interactions with groups of people.

### 2.1. Interaction Dynamics Within Collaborative Teams of People

We first take a look at prior work examining the social dynamics of psychological safety and inclusion as well as the role of backchanneling within human teams.

#### 2.1.1. Team Social Dynamics: Psychological Safety and Inclusion

Psychological safety is a term coined by Amy Edmondson and is defined as a “shared belief held by members of a team that the team is safe for interpersonal risk taking” (Edmondson, 1999). Edmondson (1999) demonstrated that psychological safety does positively influence team performance, and that the relationship between the two is moderated by the team's learning behavior (e.g., asking for help, seeking feedback, and discussing errors). Psychological safety has also been shown to positively correlate with leader inclusiveness (Nembhard and Edmondson, 2006), team member engagement in quality improvement efforts (Nembhard and Edmondson, 2006), high-quality relationships (Carmeli et al., 2009), a more positive attitude about teamwork (Ulloa and Adams, 2004), and both exploratory and exploitative learning (Kostopoulos and Bozionelos, 2011). Additionally, a comprehensive survey at Google, involving over 200 interviews and examining hundreds of attributes of more than 180 Google teams, concluded that psychological safety was the most influential factor in the success of Google teams (Rozovsky, 2015).

In addition to psychological safety, inclusion is also an important contributor to the success and productivity of collaborative teams (Oswick and Noon, 2014). Building and maintaining an inclusive environment within a team can be challenging due to the ease at which subgroups and intergroup biases form within a group (Dunham et al., 2011). Intergroup biases result in an “us vs. them” or an ingroup and outgroup perspective, driving members to act in ways that fortify ingroup-outgroup divides (Baron and Dunham, 2015). However, teams can grow in inclusion by valuing the unique contributions of each team member and conveying to each team member that they belong on the team (Shore et al., 2011). Teams that achieve a high level of inclusion result in higher team member commitment as well as greater performance outcomes (Cho and Mor Barak, 2008; Shore et al., 2011; Sabharwal, 2014).

In this work, we seek to improve the psychological safety and inclusion of human team members through the verbal support of a robot. In our experimental design, we form a 2 person ingroup and 1 person outgroup, and are especially interested in how robot verbal support might improve the inclusion and psychological safety of the outgroup member.

#### 2.1.2. Backchanneling in Human Teams

While psychological safety and inclusion can be regarded as high-level characteristics of teams, the backchanneling behavior of team members is a lower-level interaction dynamic that is also important for team success. A study by Jung et al. (2012) showed that pair programmers who exhibited more backchanneling while completing a collaborative task demonstrated higher objective performance scores as well as higher satisfaction ratings of their work and the overall experience. Backchannels, as defined by Ward and Tsukahara (2000), are “the short utterances produced by one participant in a conversation while the other is talking.” This backchanneling feedback occurs regularly and frequently in conversation. The Japanese language even has a term, *aizuchi*, to describe verbal backchannels, which occur frequently in conversation and are sometimes even actively elicited. With respect to American English, one study found that 19% of all utterances consisted of some form of verbal backchanneling (Jurafsky et al., 1997). Backchannel responses are often not limited to just verbal utterances but also consist of non-verbal signals such as head nodding and shaking (Duncan, 1974; Stubbe, 1998).

Backchannels confirm that the “speaker and listener share a common frame of reference” without taking a speaker's conversational turn (Duncan, 1974) or threatening the speaker's position as primary speaker (Stubbe, 1998). Goodwin (1986) highlights two specific functions of backchannels: (1) to encourage the speaker to continue talking (e.g., a backchannel of “uh huh” in the middle of a speaker's continued speech) and (2) acknowledges and briefly assesses the speech of the speaker (e.g., a backchannel of “oh wow” indicating both acknowledgement and surprise). Backchanneling has been shown to occur more frequently in conversations where people get to know one another as opposed to competitive debates (Dixon and Foster, 1998). Additionally, several studies have found that females backchannel more frequently than males (Roger and Nesshoever, 1987; Duncan and Fiske, 2015).

While Jung et al. (2012) have shown a connection between the presence of backchanneling behavior with task performance and team member satisfaction, in this work, we seek to understand how the backchanneling behavior of human team members may connect with the social dynamics of psychological safety and inclusion. We also seek to investigate how verbal support from a robot might influence the backchanneling behavior of the robot's human team members.

### 2.2. Robot Interactions With Groups and Teams of People

Now that we have taken stock of prior work examining social dynamics and interactions within human teams, we focus our attention on the growing body of work that investigates robot social interactions with groups and teams of people as well as work focusing on robot backchanneling.

#### 2.2.1. Robots That Shape Group Behavior and Dynamics

There has been an increasing focus in the field of human-robot interaction (HRI) on developing robots that can seamlessly and intelligently interact with multiple people, see Sebo et al., 2020 for a review. Some work in HRI has examined how a robot's physical movements such as navigation (Kidokoro et al., 2013; Mavrogiannis et al., 2019), physical orientation (Shiomi et al., 2010; Vázquez et al., 2017), gestures (Liu et al., 2013; Hoffman et al., 2015), and gaze (Mutlu et al., 2009; Skantze, 2017) can improve human-robot group interactions and influence people's perceptions of the group. Other work in HRI has explored how a robot's verbal utterances that convey expressions of emotion (Leite et al., 2012; Correia et al., 2018), informational content (Sabelli and Kanda, 2016; Fernández-Llamas et al., 2017), and the robot's personality (Kanda et al., 2012; Oliveira et al., 2018) can shape people's perceptions of the group and the robot as well as build social relationships between the robot and the people with whom it interacts.

Beyond the work that has focused on how robot behavior can shape people's perceptions of the robot and of the group as a whole, additional work within HRI has focused on how robot actions can influence human-to-human interactions within a group. Robots have been shown to increase interactions between people in elder care facilities (Šabanović et al., 2013; Thompson et al., 2017), between the members of inter-generational groups (Short et al., 2017; Joshi and Šabanović, 2019), and between children with ASD and those with whom they interact (Kim et al., 2013; Zubrycki and Granosik, 2016; Scassellati et al., 2018). Robots have also demonstrated success in moderating conflict (Shen et al., 2018) and raising awareness to a team that conflict has occurred (Jung et al., 2015). Through different moderation strategies, a robot was able to shape human members' perceptions of the group's cohesion (Short and Matarić, 2017). A simple microphone robot has displayed success in facilitating a more balanced participation during a three-person team's decision making discussion (Tennent et al., 2019). Lastly, a robot's verbal expressions of vulnerability caused “ripple effects” in a group by increasing how likely human members of the group are vulnerable with one another (Strohkorb Sebo et al., 2018).

Although this work has contributed greatly to our understanding of how robot verbal behavior can influence human-robot groups, little work has explored how robot behavior might be able to shape the important social dynamics of psychological safety and inclusion within human-robot teams.

#### 2.2.2. Robots Backchanneling in Human-Robot Interactions

In order to promote inclusion and psychological safety in human-robot teams, in this work we investigate the use of verbal support from a robot. The verbal support we employ predominately comes in the form of verbal backchannels, which have been studied to a growing degree within the HRI community.

In order to increase the quality of communicative interactions and to encourage positive behavior from the humans with which they interact, HRI researchers have incorporated backchanneling behaviors in human-robot interactions (Lala et al., 2017; Ramachandran et al., 2018). For example, Ramachandran et al. (2018) designed a tutoring robot to display the non-verbal backchannel of head nodding while a child responded to one of the robot's prompts. Additionally, Lee et al. (2019) designed an attentive listening behavior generation model for a robot in a child storytelling context, which they demonstrated to be more effective than an approach based on signaling. Other work has demonstrated the utility of backchannels from a robot in human-robot collaborative teaming. Jung et al. (2013) demonstrated that the presence of robot backchannels led to improved team functioning, where the presence of robot backchanneling was correlated with increased performance (decreased reaction time) as well as reduced human stress and increased perceptions of responsiveness in high complexity tasks. This work investigating the use of robot backchannels, especially Jung et al. (2013), suggests that verbal support from a robot has the potential to positively influence the psychological safety and inclusion of human team members.

## 3. Materials and Methods

In this section, we describe the human subjects experiment we designed to study the effects of robot verbal support on the social dynamics and interactions within a human-robot team.

### 3.1. Experimental Design

**Figure 2:**
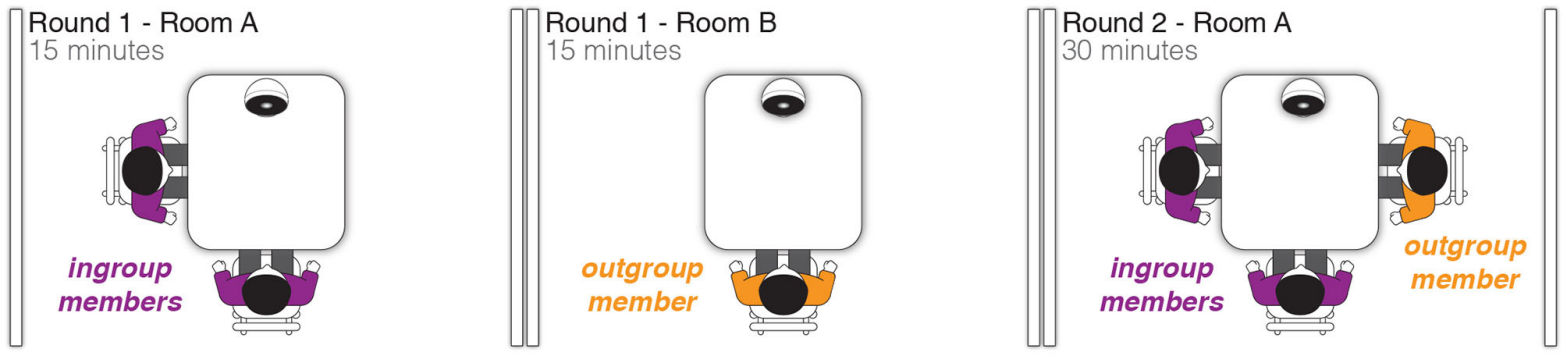
We formed an intergroup bias between our three participants in the first round of the task by having two participants (ingroup) and a robot work together in room A and the other one participant (outgroup) and a robot work in room B. Then for the second round of the task, the outgroup participant joined the two ingroup participants and the robot in room A.

This experiment had a between-subjects design where three participants took part in a collaborative task and either (1) interacted with a robot that gave them verbal support or (2) interacted with a robot that did not give them verbal support. We were especially interested in how the verbal support from the robot might influence an outgroup member. In both of our between subject conditions, we formed an ingroup-outgroup division within each group of three participants through two rounds of a collaborative task, see Figure 2.

In the first round, which lasted 15 min, two participants and a robot worked together on a task in room A. In room B, the third participant and a robot completed the same task. This first round was designed to form an ingroup, consisting of the two participants in room A, and an outgroup, the one participant in room B. Then for the second round, which lasted for 30 min, the outgroup participant was brought into room A to join the two ingroup participants and the robot. All three participants and the robot worked together to complete the round two task.

### 3.2. Collaborative Task

Participants in our experiment collaborated with one another and the robot to complete a modified version of the Desert Survival Problem (Lafferty and Pond, 1974). This task had two rounds. In the first round, participants were given 15 min to rank 25 common household items (e.g., chocolate, umbrella, rubber bands) with respect to how useful they are for survival. Participants could ask the robot for more information about each item by verbally querying the robot. For example, a participant query about the watch was met with a robot response of, “*this Rolex is covered in 24 carat gold and contains a shiny diamond in the center, the batteries should last for about 5 years*.” All information required for participants to query the robot (how to query the robot, which items they can query the robot about) was given to them on an instruction sheet at the beginning of this first round.

In the second round, participants were given 30 min to select and rank 8 items from the same list of 25 common household items they were given in the first round. Participants could also ask the robot for information about the environment where they were to be stranded (e.g., weather, temperature, wildlife) and they were given an updated instruction sheet. For example, when participants queried the robot about the water supply, the robot responded, “*there is one running stream of water 15 miles from where you are stranded*.” This additional information about the environment in the second round was designed to encourage participants to question prior assumptions and spark further discussion.

### 3.3. Robot Behavior

We used the commercial robot Jibo for this experiment (Jibo, 2017). Jibo is 11 inches tall and has a 3-axis motor system and a touchscreen face. We enabled Jibo to respond verbally to the participant utterances by capturing the participant's audio through individual microphones and using Google's speech-to-text API to acquire the spoken text.

**Figure 3:**
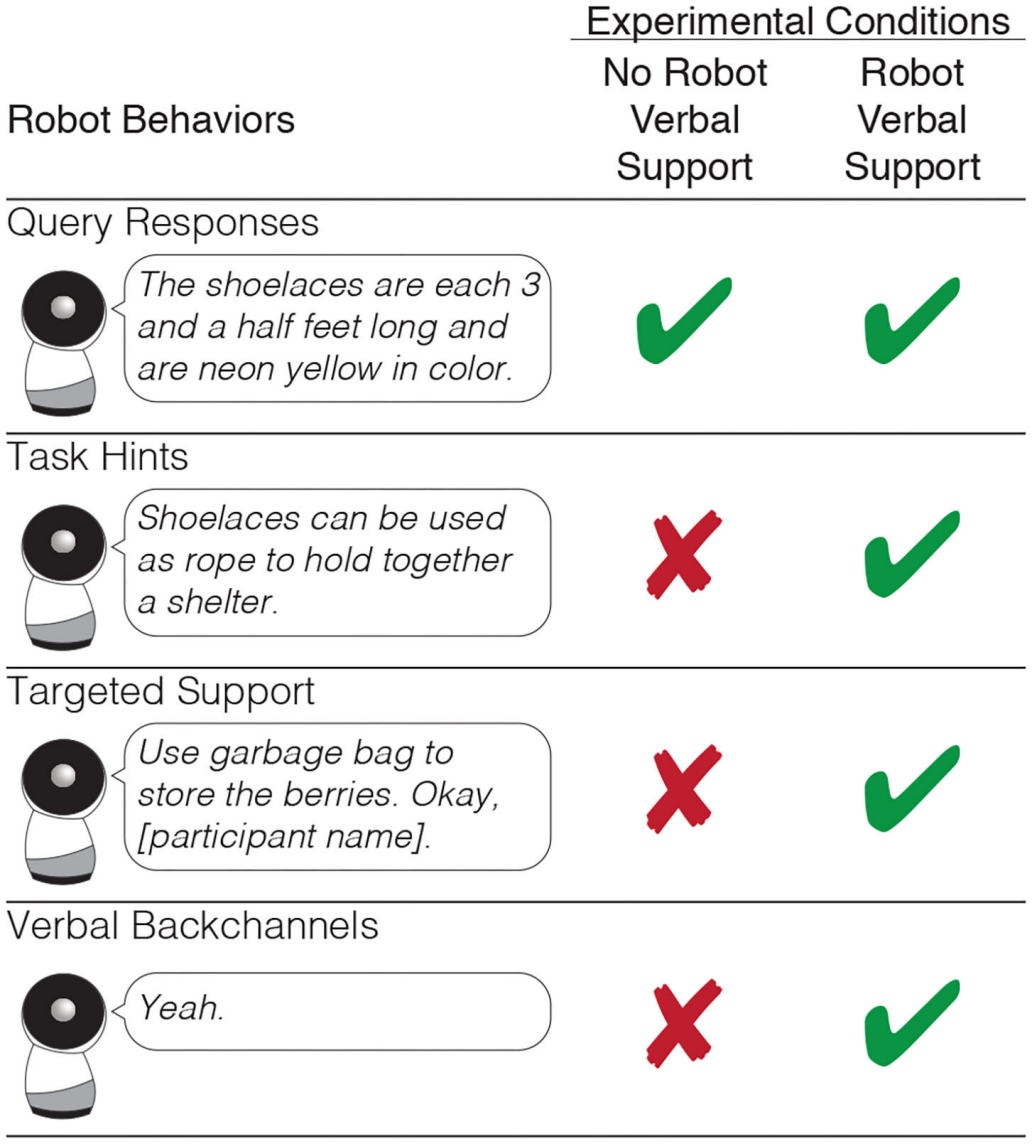
Our experiment contained two between subjects conditions defined by either the presence or absence of verbal support from the robot that came in the form of task hints, targeted support, and verbal backchannels. In both conditions, the robot responded to queries for information from the participants.

During the collaborative task, the robot made several different types of verbal utterances: query responses, task hints, targeted support, and verbal backchannels. In the conditions where the robot *did not* give verbal support, the robot made only query responses. In the conditions where the robot *did* give verbal support, the robot made all of the aforementioned verbal utterances, see Figure 3. In the robot verbal support conditions, the robot's verbal utterances were *equally distributed* between the three participants.

There was often a delay of a few seconds in between the end of a participant utterance and the start of the robot's verbal response due to the time it took to get the speech-to-text transcript from the Google server and the latency of the robot executing the utterance. Most of the time, this was not a problem, because participants normally waited for Jibo's query responses and Jibo's verbal backchannels (e.g., “*mm hmm*”) did not take the conversational floor. However, sometimes the delay in Jibo's response did interrupt the participants.

#### 3.3.1. Query Responses

Querying the robot was essential for participants to have the full information to complete the task. Participants could query the robot about the survival items in both the first and second rounds as well as aspects about the environment during the second round using the language, “hey Jibo, tell me about the ____,” where Jibo is the name of the robot. During the second round, only the participant who was designated as the robot liaison could query the robot. We introduced this robot liaison role in order to study the effects of this role on perceived inclusion of participants. The results of that analysis can be found in Strohkorb Sebo et al. (2020). In this work, we do not focus specifically on the robot liaison role, however, we do statistically control for it in our analysis by adding it as a covariate in our statistical models.

Queries about the survival items gave participants more detailed information about the quantity and type of the item, for example, when queried about the chocolate the robot responded with, “*this box comes with 16 bars of 17.6 ounces Trader Joe's chocolate. Each bar is wrapped in tinfoil and then with paper*.” Queries about environment aspects provided participants with information that was designed to stimulate conversation and have participants question prior assumptions they may have made about the location in which they were stranded. For example, the robot responded to being queried about the geography with: “*The whole area is one big mountain range. Some of the mountains might be covered in snow while others are more temperate and covered with grass. You may come upon some caves and lowlands as well*.” During the second round of the collaborative task, the robot made 18.22 (*SD* = 7.77) query responses on average to participants in the robot verbal support condition and 20.10 (*SD* = 5.40) query responses on average to participants in the no robot verbal support condition.

#### 3.3.2. Task Hints

After hearing a participant mention a survival item name, outside the context of a query to the robot, we had the robot deliver a useful hint about the survival item with a fixed probability. The survival item hints were designed to encourage the participants to consider alternative uses, for example, the robot's hints about the garbage bag included: “*a garbage bag can be used as a sleeping bag*” and “*garbage bags can collect rain water*.” Participants in the robot verbal support condition received an average of 1.30 (*SD* = 1.03) task hints from the robot in the second round of the collaborative task.

#### 3.3.3. Targeted Support

We programmed the robot to deliver six targeted supportive utterances to each participant during the second round of the task. We designed the targeted supportive utterances to positively reinforce ideas and viewpoints of specific participants and to be personal by including the participant's name. Targeted supportive utterances either rephrased what a participant said (e.g., “*We need a coffee pot, good idea Samantha*”), included an item that a participant had mentioned (e.g., “*Camera. Robert, I think that's worth considering*”), or gave general support for the participant (e.g., “*Okay, Jason*”). Participants in the robot verbal support condition received an average of 16.33 (*SD* = 2.40) targeted supportive utterances from the robot in the second round of the collaborative task, spread evenly between the three participants.

#### 3.3.4. Verbal Backchannels

Lastly, after hearing any participant utterance, the robot responded with a backchannel utterance with a fixed probability. If the participant's speech contained the mention of one of the survival items, the robot might respond with an item backchannel (e.g., “*balloon, that makes sense*,” “*key, uh huh*”). If the participant's speech did not contain a survival item, the robot responded with a generic backchannel (e.g., “*yeah*,” “*interesting*,” “*hmm*”). The robot's verbal backchannels were either positive in nature (e.g., “*yeah*”) or neutral (e.g., “*hmm*”), but were never negative. Participants in the robot verbal support condition received an average of 34.04 (*SD* = 11.73) verbal backchannels from the robot in the second round of the collaborative task.

### 3.4. Protocol

For each experimental session, we recruited three human participants. Each participant, and a parent/guardian if the participant was under the age of 18, completed a consent form prior to their participation in the experiment. After all three participants arrived, they each filled out a pre-experiment questionnaire on a tablet. To get the participants set up for the first round of the task, the experimenter took the outgroup participant to room B with one Jibo robot, ensured that the participant could query the robot properly, and initiated the robot's introduction to round 1. While the experimenter was setting up the outgroup participant, the two ingroup participants were instructed to ask one another questions from a list of get-to-know-you questions in order to further reinforce the ingroup-outgroup divide (e.g., “If you didn't sleep, what would you do with your extra time?”). After the experimenter had set up the outgroup participant, the experimenter paused the ingroup members and set them up in room A similarly to the outgroup participant.

After the 15 min of round 1 had expired, the experimenter brought the outgroup participant into room A with the two ingroup participants and the Jibo robot for round 2 of the collaborative task. The experimenter then designated one of the three participants as the robot liaison using the language, “*In this part, unlike the first, only one of you will be able to ask Jibo questions about the items and environment. For all of you this is [participant name]*.” The robot liaison was chosen randomly before the study took place. For half of the groups, the robot liaison was an outgroup member and for the other half, the robot liaison was an ingroup member. Round 2 concluded after 30 min. After that, the experimenter brought the three participants out of room A and administered the post-experiment questionnaire to the participants on tablets. After each participant completed their post-experiment questionnaire, they were compensated with $10.

### 3.5. Measures

In order to test the relationships between participant backchanneling behavior, their ratings of the team's social dynamics, and the robot's verbal support, we detail the questionnaire measures we administered to the participants as well as our annotation of participants' backchannels.

#### 3.5.1. Controls

In order to assess pre-existing differences between participants that might influence their behavior in this experiment, in the pre-experiment survey we measured participants' prior familiarity with the other two participants, their extraversion, and their emotional intelligence. We captured participants' prior familiarity with the other human participants because a high prior familiarity is likely to positively correlate with higher ratings of inclusion and psychological safety. We collected participants' ratings of their extraversion because it is a needed covariate when analyzing the data related to the amount of time that participants' spend talking. Finally, we assessed participants' emotional intelligence because prior work has found positive correlations between team member emotional intelligence and team perceptions of psychological safety (Harper and White, 2013).

Participants evaluated their prior familiarity with the other two human participants in the group by choosing the most appropriate descriptor for their relationship from 1 (*I have not met this participant before we completed this study together; I do not know them*) to 5 (*I would consider this participant to be one of my closest friends*) as well as denoting whether they had each others' phone numbers and whether they were connected on social media. Participants also rated their extraversion according to the abbreviated version of the Revised Eysenck Personality Questionnaire (EPQR-A) from Francis et al. (1992) and their emotional intelligence according to the Short Form of the Trait Emotional Intelligence Questionnaire (TEIQue-SF) from Cooper and Petrides (2010).

#### 3.5.2. Perceived Group Dynamics

In the post-experiment survey, participants completed the Perceived Group Inclusion Scale (Jansen et al., 2014), a 16 item scale that asked participants to evaluate items such as “this group gives me the feeling that I belong” and “this group encourages me to be authentic” on a Likert scale from 1 (*Strongly Disagree*) to 5 (*Strongly Agree*).

Participants also filled out the Team Psychological Safety Scale (Edmondson, 1999), a 7 item scale where participants rated their agreement to statements like “it is safe to take a risk on this team” and “members of this team are able to bring up problems and tough issues” on a Likert scale from 1 (*Strongly Disagree*) to 7 (*Strongly Agree*).

#### 3.5.3. Perceptions of the Robot

In order to capture participants' perceptions of the robot, participants completed the Robotic Social Attributes Scale (RoSAS) from Carpinella et al. (2017) in the post-experiment survey. The RoSAS scale has three dimensions (warmth, competence, and discomfort), where participants rated the robot on 6 traits within each dimension on a 9 point Likert scale from 1 (*Definitely Not Associated*) to 9 (*Definitely Associated*).

#### 3.5.4. Human Speech

Each human participant wore a headset microphone throughout the experiment. The participants' audio data was transcribed using Google's speech-to-text API. During the experiment, we fed the Google speech-to-text transcripts to the tablet controlling the robot in order to allow the robot to respond to participants' speech. Additionally, we stored these speech-to-text transcriptions as well as the start time and duration of the speech, so that we could measure how much time each participant spent talking over the course of the experiment.

#### 3.5.5. Human Backchannels

In order to analyze the backchanneling behavior of the human participants, we transcribed and categorized each backchannel made by the participants during round 2 of the experiment using the ELAN software (Wittenburg et al., 2006). We used the backchannel definition from Ward and Tsukahara (2000) to distinguish between backchannels and non-backchannel utterances: “backchannel feedback (1) responds directly to the content of an utterance of the other, (2) is optional, and (3) does not require acknowledgement by the other.” Each backchannel was categorized as either verbal (e.g., “*okay*,” “*mm hmm*,” “*yeah yeah*”) or non-verbal (e.g., head nodding, head shaking). Each backchannel was also annotated with a recipient, indicating to whom the backchannel was directed toward.

Four coders contributed to the identification and categorization of human backchannels in the data. Inter-rater reliability was assessed by examining the agreement of the coders on candidate backchannels (a high Cohen's kappa value of 0.90) and on the backchannel recipient (a high Cohen's kappa value of 0.93). Participants on average produced 27.06 (*SD* = 21.41) non-verbal backchannels and 30.67 (*SD* = 15.85) verbal backchannels during the 28 annotated minutes of round 2.

### 3.6. Participants

Participants were recruited for this study from a high school program held at Yale University, where over 50% of the attendees were international students. A total of 40 groups (120 participants) were recruited for participation in this study. Of the 40 groups recruited, 1 group was excluded from this analysis because they did not finish the experimental task. For the 39 remaining groups (117 participants), 59 participants were female and 58 participants were male. The average age of participants was 16.73 (*SD* = 0.72).

In the robot verbal support condition, there were 87 participants (29 groups) with an average age of 16.77 (*SD* = 0.73) and with 42 female and 45 male participants. In the no robot verbal support condition, there were 30 participants (10 groups) with an average age of 16.60 (*SD* = 0.72) and with 17 female and 13 male participants.

## 4. Results

We used linear mixed-effects models in the analysis of our data in order to account for participants being in groups of three. We set the variables related to our experimental manipulations as fixed effects: intergroup bias (ingroup or outgroup), robot verbal support (yes or no), and interactions between those variables. We also set relevant covariates as fixed effects: robot liaison designation (yes or no), gender, extraversion, emotional intelligence, and familiarity with other human team members. We set the participant's group as a random effect (random intercept) and relevant covariates as fixed effects. We tested these models for multicollinearity (variance inflation factor), selected them based on the Akaike information criterion, and evaluated residual errors for lack of trends and heteroscedasticity. For each fixed effect, the model outputs the linear coefficient (*c*), the standard error (*SE*), and the significance (*p*) value of that predictor.

When analyzing data where each data point represented one group of three participants, we used an analysis of variance (ANOVA) test. We investigated the influence of effects of intergroup bias (ingroup or outgroup), robot verbal support (yes or no), and several covariates (robot liaison designation, gender, extraversion, emotional intelligence, and familiarity with other human team members) on our dependent variables of interest. We report the effect size as partial eta squared (η^2^).

### 4.1. Participant Responses to Verbal Support From the Robot

For the 29 groups (87 participants) who received verbal support from the robot, we analyzed how participants responded to the targeted support they received from the robot. We were especially interested in whether the one outgroup member, compared with the two ingroup members, would benefit more or less from this targeted support from the robot. Each participant received, on average, 5.62 (*SD* = 0.86, min = 2, max = 7) targeted supportive utterances during the collaborative task. Eighty-one (93%) of the participants received 5–7 targeted supportive utterances, as we intended. However, 6 (7%) of the participants received 2–4 targeted supportive utterances due to their long periods of silence, since the robot's targeted supportive utterances were only triggered after a participant spoke. These targeted supportive utterances from the robot supported the ideas and verbal comments made by participants, including the participant's name in the utterance (e.g., “*Tape is useful in any kind of situation; makes sense to me, Anthony*”).

In order to examine participant responses to the targeted supportive utterances they received from the robot, we examined the proportion of time participants spoke in the 1 min after they received the targeted support from the robot (*robot support targeted to participant - RST-P*). We compared participant responses to targeted support from the robot with two controls: (1) the proportion of time participants spent talking in the one minute after the robot made a targeted supportive utterance to someone else (*robot support targeted to someone else - RST-SE*) and (2) the proportion of time participants spent talking in the one minute after the robot made an undirected utterance (*robot undirected utterance - RUU*), such as a general backchannel (e.g., “*yeah*,” “*okay*”). We excluded the data from 13 participants (from 6 different groups) from this analysis due to the following reasons: participant silence for the entire 30 min of part 2 of the experiment (*n* = 1), participant non-compliance by removing the microphone (*n* = 4), microphone disconnection (*n* = 3), and errors in logging the participant speech data (*n* = 6).

**Figure 4:**
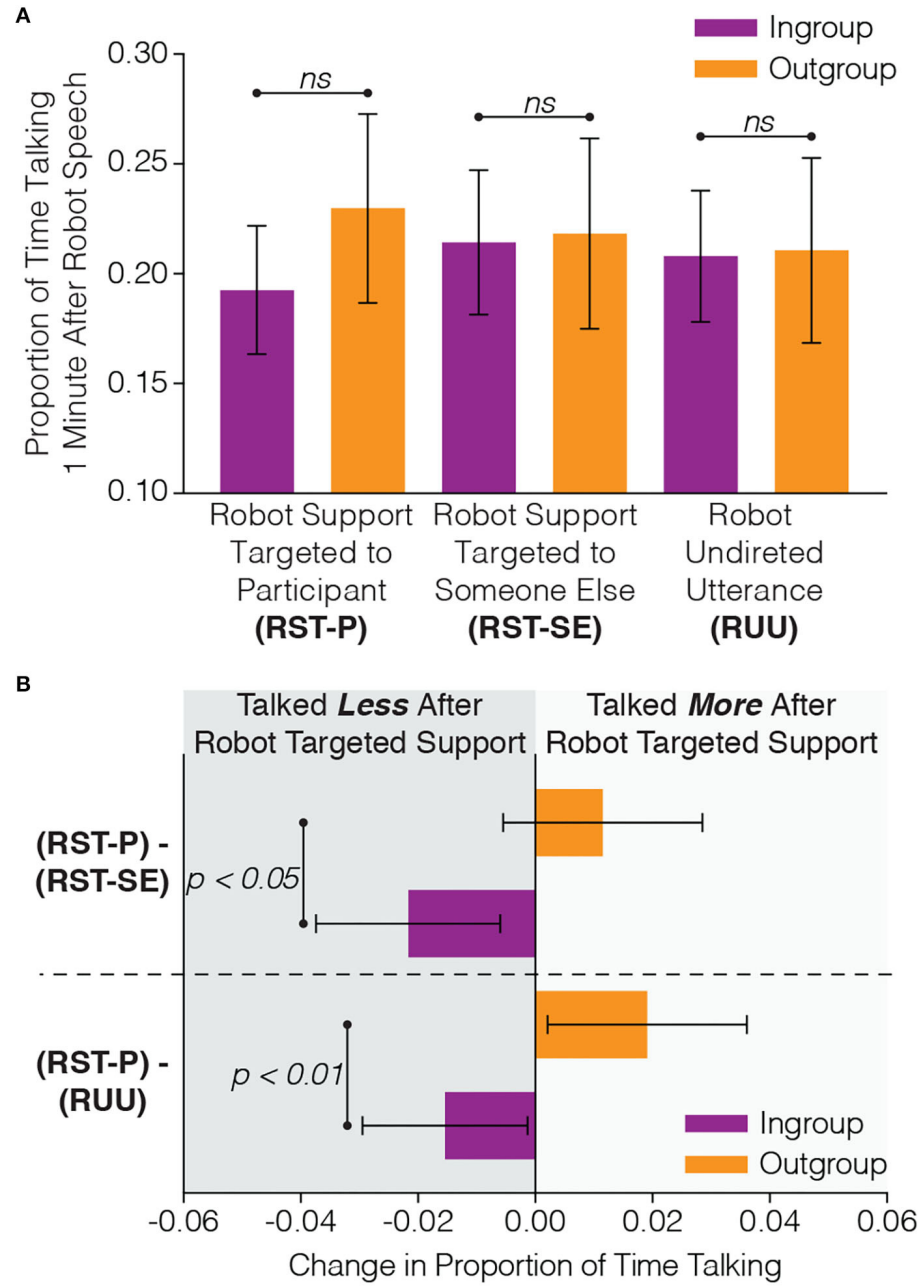
In order to determine how participants responded to the targeted robot support (e.g., “*Tape, good idea Matthew*”), we examined how much participants talked in the 1 min after robot support targeted to the participant (RST-P) and two controls: (1) the amount of time participants spent talking after robot support targeted to someone else (RST-SE) and (2) robot undirected utterances (RUU). Although there were **(A)** no significant differences between ingroup and outgroup members on their responses to the robot utterances, **(B)** the difference in the amount of response to the robot support targeted to the participant (RST-P) compared with the two controls (RST-SE and RUU) revealed significant differences between ingroup and outgroup members where outgroup members demonstrated a greater increase in talking time after the robot targeted them with verbal support.

As shown in Figure 4A, we did not find a significant difference in the proportion of time that participants spoke during the 1 min after robot targeted support to the participant, RST-P, (*c* = 0.01, *SE* = 0.03, *p* = 0.703), during the 1 min after robot targeted support to someone else, RST-SE, (*c* = −0.03, *SE* = 0.03, *p* = 0.445), or during the 1 min after robot undirected utterances, RUU, (*c* = −0.03, *SE* = 0.03, *p* = 0.263).

However, when we analyzed the *difference* between the proportion of time participants spent talking during the 1 min after they received targeted support from the robot (RST-P) with our two controls (RTS-SE and RUU), we did find significant differences between the ingroup and outgroup participants, see Figure 4B. When examining the difference in the proportion of time participants talked during the 1 min after the robot gave them targeted support with the proportion of time participants talked during after the robot gave someone else targeted support (RST-P − RST-SE), we found that outgroup members (*M* = 0.012, *SD* = 0.041) had significantly higher difference values than ingroup members (*M* = −0.022, *SD* = 0.055, *c* = 0.03, *SE* = 0.02, *p* = 0.047). This demonstrates that outgroup members, as opposed to ingroup members, talked more after the robot gave them targeted support than when the robot gave someone else targeted support. We found the same result when analyzing the difference in the proportion of time participants talked during the 1 min after the robot gave them targeted support with the proportion of time participants talked during after the robot made an undirected utterance (RST-P − RUU). Again, outgroup members (*M* = 0.019, *SD* = 0.041) had significantly higher difference values than ingroup members (*M* = −0.015, *SD* = 0.049, *c* = 0.04, *SE* = 0.02, *p* = 0.007), emphasizing that outgroup members had a larger response in talking time to robot targeted support when compared with other utterances made by the robot. These results indicate that targeted support from the robot, as opposed to other utterances the robot might make, are especially effective at encouraging verbal contribution from human outgroup members.

### 4.2. The Influence of Robot Verbal Support on Human Verbal Support

Based on prior work that demonstrated a positive correlation between the presence of backchanneling (e.g., “*yeah*,” head nodding) and team performance (Jung et al., 2012), we investigated the influence of the verbal support from the robot on the support (backchanneling) the humans in the group gave one another. First, we analyzed correlations between the verbal (e.g., “*yeah*,” “*mm hmm*”) and non-verbal backchannels (e.g., head nodding) we annotated from the data with participants' self-reported psychological safety and inclusion scores. Then, we examined the influence of robot verbal support on the human-to-human backchannel support that was most significantly related to participants' ratings of psychological safety and inclusion.

#### 4.2.1. Correlations Between Participants' Backchannels and Their Ratings of Team Social Dynamics

**Figure 5:**
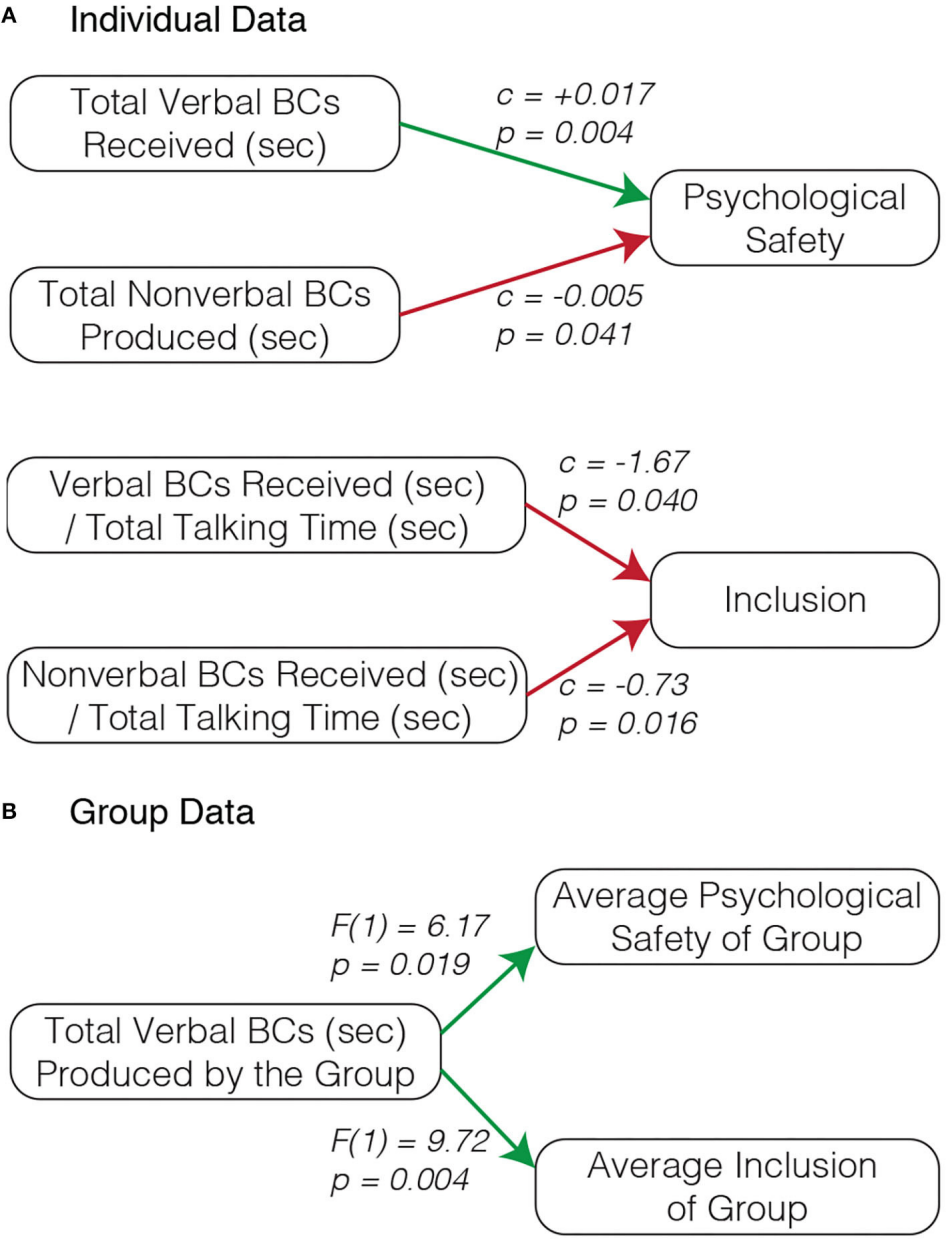
Analysis of human participant backchannels (BCs) within the groups demonstrated that certain backchannel features predicted the psychological safety and inclusion scores of the participants. By examining each individual participant's data, we found that **(A)** the total amount of verbal backchannels received (sec) positively correlates with participants' psychological safety scores, the total amount of non-verbal backchannels produced (sec) negatively correlates with participants' psychological safety scores, and both the non-verbal and verbal backchannels (sec) received normalized by the participant's talking time negatively correlates with participants' inclusion scores. When considering each group, we found that **(B)** the total amount of verbal backchannels produced by the group positively correlates with both the group's average psychological safety and inclusion scores. Green arrows indicate positive correlations and red arrows indicate negative correlations.

We first performed an analysis examining correlations between participant backchannels and their ratings of team social dynamics where each participant represents one data point. We excluded the data from 1 group (3 participants) because 2 of the 3 participants in this group did not comply with wearing their microphone. We found several significant correlations between the backchanneling behavior of participants and their reported psychological safety and inclusion scores, see Figure 5A.

We found a significant positive influence of the total amount of verbal backchannels a participant received (sec) on their psychological safety score (*c* = 0.017, *SE* = 0.006, *p* = 0.004), indicating that a person who receives a large amount of verbal backchannels is also likely to have a high psychological safety score. We also found that psychological safety scores were correlated negatively with the total time a participant produced non-verbal backchannels (*c* = −0.0047, *SE* = 0.0023, *p* = 0.041), meaning that participants who produced a lot of non-verbal backchannels (head nodding) had lower psychological safety ratings than those who did not express as many non-verbal backchannels.

With respect to participants' inclusion scores, we found that they were influenced by both the amount of time participants received verbal and non-verbal backchannels normalized by the total time that a participant spent talking, in other words, the proportion of the time a participant spent talking where they were being backchanneled by others. We discovered a significant negative correlation between both the amount of time participants received *verbal* backchannels normalized by their total talking time (*c* = −1.67, *SE* = 0.80, *p* = 0.040) and the amount of time participants received *non-verbal* backchannels normalized by their total talking time (*c* = −0.73, *SE* = 0.30, *p* = 0.016) with participants inclusions scores. It is interesting to consider these findings together with the results that indicate that the amount of verbal backchannels received is positively correlated with psychological safety. Although backchanneling another person in a group may raise their psychological safety, backchanneling them too much relative to their talking time may result in reduced inclusion. It is also possible that teams with conversationally dominant members may give the dominant member a higher proportion of backchannels and, as a result of the conversationally dominant member, report reduced inclusion scores.

We also examined the data by considering each group as one data point. We computed the total amount of backchanneling that occurred in each group and we averaged their psychological safety and inclusion scores. As shown in Figure 5B, We found that the time participants within each group spent verbally backchanneling one another had a significant and positive influence on both groups' average inclusion scores, $F_{\left(1\right)}=9.72,\eta^{2}=0.11,p=0.004$, as well as groups' average psychological safety scores, $F_{\left(1\right)}=6.17,\eta^{2}=0.038,p=0.019$. These results indicate a similar finding to what we observed with the individual data: the total volume of verbal backchannels a participant received correlates with more positive perceptions of team social dynamics. Though, with individuals this relationship was only significant between the verbal backchannels a person received and their psychological safety score, groups who had more verbal backchannels toward one another had higher psychological safety scores *and* inclusion scores.

#### 4.2.2. The Effects of Robot Verbal Support on the Verbal Backchannels a Participant Received

From our analysis of the correlations between participants' backchanneling behavior and important team social dynamics, psychological safety and inclusion, we saw that verbal backchanneling is predictive of both team members' psychological safety and inclusion scores. We then examined whether the verbal support from the robot influenced the verbal backchanneling of human team members. Just as in section 4.2.1, we excluded the data from 1 group (3 participants) because 2 of the 3 participants in this group did not comply with wearing their microphone.

**Figure 6:**
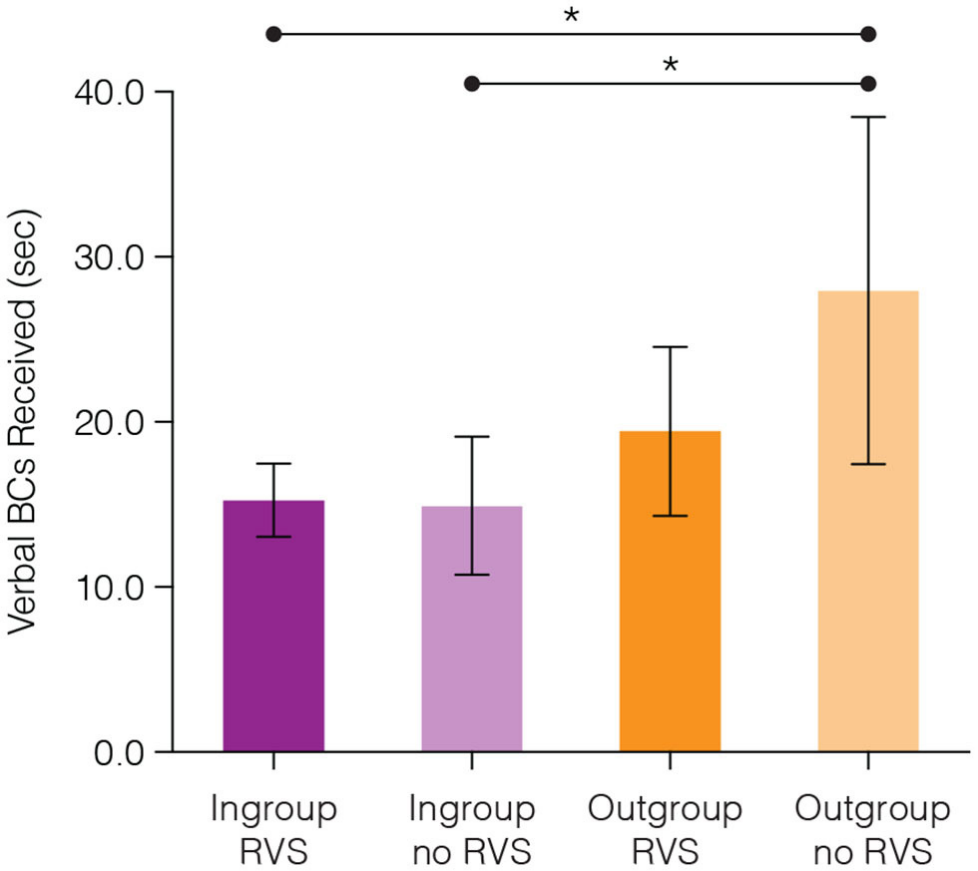
Outgroup members who were in a group with no robot verbal support (no RVS) received significantly more verbal backchannels (sec) from their human teammates than ingroup members in groups with robot verbal support (RVS) and without robot verbal support. *p < 0.05 and error bars represent a 95% confidence interval.

We found a significant influence of intergroup bias (ingroup-outgroup membership) on the amount of verbal backchannels a participant received, where outgroup members received more verbal backchannels (*M* = 21.68*s, SD* = 13.92*s*) than ingroup members (*M* = 15.17*s, SD* = 8.39*s, c* = 16.85, *SE* = 4.09, *p* < 0.001). We also found a significant interaction between intergroup bias and the presence of verbal support from the robot (*c* = −8.83, *SE* = 4.33, *p* = 0.045), shown in Figure 6. Using *post-hoc* comparisons using Tukey-adjusted estimated marginal means, we found that outgroup members with no robot verbal support received significantly more verbal backchannels (*M* = 27.96*s, SD* = 14.71*s*) than both ingroup members with robot verbal support (*M* = 15.26, *SD* = 8.28, *c* = −10.72, *SE* = 3.44, *p* = 0.013) and ingroup members with no robot verbal support (*M* = 14.92, *SD* = 8.93, *c* = −11.29, *SE* = 3.78, *p* = 0.019). No other comparisons significantly differed from one another.

This interaction effect seems to be primarily driven by the higher amount of backchanneling received by the outgroup member in groups with no robot verbal support. Without the presence of robot verbal support outgroup members received significantly more backchannels than ingroup members. When there is robot verbal support present, we did not find a significant difference between the backchannels received by ingroup and outgroup members. This result suggests that when the robot exhibits more verbal support, the ingroup members may not see the need to backchannel the outgroup member quite as much.

It is possible that the amount of time spent talking may have influenced this result, where those who talk more would likely receive more backchannels from their peers. Therefore, we examined the influence of intergroup bias (ingroup-outgroup membership) and the presence of verbal support from the robot on the amount of time participants spent talking in the experiment. We did not find any significant effects of intergroup bias (*c* = −17.58, *SE* = 57.39, *p* = 0.760), the presence of verbal support from the robot (*c* = −49.78, *SE* = 43.27, *p* = 254), or the interaction between these two variables (*c* = −4.83, *SE* = 67.69, *p* = 0.943) on the amount of time participants spent talking during the experiment. Therefore, although intergroup bias and robot verbal support *did not* influence the amount participants spoke during the experiment, intergroup bias and robot verbal support *did* influence how much verbal backchanneling participants received during the experiment.

### 4.3. The Effects of Robot Verbal Support on Participant Ratings of Psychological Safety and Inclusion

We also examined the effect of the presence of robot verbal support on the psychological safety and perceived inclusion scores of participants. We found a marginally significant effect of robot verbal support on participants' psychological safety scores (*c* = 0.35, *SE* = 0.18, *p* = 0.056), where participants in groups with robot verbal support had slightly higher psychological safety scores (*M* = 6.06, *SD* = 0.74) than participants in groups without robot verbal support (*M* = 5.92, *SD* = 0.74). We did not find any significant differences in participants' perceived inclusion scores between those who experienced robot verbal support (*M* = 4.32, *SD* = 0.57) and those who did not experience robot verbal support (*M* = 4.34, *SD* = 0.61, *c* = 0.12, *SE* = 0.14, *p* = 0.376). From the lack of statistically significant differences in team social dynamics scores between conditions with and without robot verbal support, we cannot make any claims that the robot verbal support in our experiment caused improvements in either participants' psychological safety or inclusion scores.

### 4.4. Perceptions of the Robot

We did not find any significant differences between participants' perceptions of the robot on the Robotic Social Attributes Scale (RoSAS) between those who did and those who did not interact with a robot that expressed verbal support. Participants who experienced verbal support from the robot had the following average ratings on the three RoSAS dimensions: warmth (*M* = 5.78, *SD* = 1.46), competence (*M* = 7.13, *SD* = 1.24), and discomfort (*M* = 2.25, *SD* = 1.10). Participants who did not experience verbal support from the robot reported the following RoSAS dimension ratings: warmth (*M* = 5.27, *SD* = 1.37), competence (*M* = 7.16, *SD* = 1.22), and discomfort (*M* = 1.74, *SD* = 0.57).

## 5. Discussion

In this work, we investigated the effects of verbal support from a robot on the social dynamics and interactions between three human team members engaged in a collaborative task. We designed two between-subjects experimental conditions, one in which a robot provided task information and another in which a robot provided both task information *and verbal support*. The verbal support from the robot came in the form of targeted support (e.g., “*whistle, good idea Tasha*”), verbal backchannels (e.g., “*yeah*,” “*okay*”), and task hints. We then analyzed how the verbal support from the robot influenced the social and interaction dynamics of all group members, with special attention paid to the one outgroup member. Here, we highlight our main findings, explore the possible mechanisms that may have led to our results, and discuss the broader implications of this work.

### 5.1. Targeted Support From the Robot Increases Outgroup Participation

For the 29 groups (87 participants) that received verbal support from the robot, we found differences between ingroup members and outgroup members in their responses to targeted support from the robot (e.g., “*Tape is useful in any kind of situation, makes sense to me Anthony*”). Outgroup members, as opposed to ingroup members, talked more after targeted support from the robot when compared with how much they talked after other types of robot utterances (Figure 4). In other words, the targeted supportive utterances from the robot were especially effective at increasing the verbal contribution from the outgroup members. It is therefore likely that employing targeted support from robots and artificial agents may be a useful strategy for overcoming intergroup bias by encouraging the verbal contributions from team members who may feel excluded.

### 5.2. Human Verbal Backchannels Positively Correlate With Psychological Safety and Inclusion Ratings

Additionally, we examined how the verbal support from the robot shaped the interactions between the human team members, specifically focusing on verbal backchannels. We identified verbal backchannels as a relevant interaction dynamic to examine through our analysis of the correlations between participants' backchanneling behavior and their ratings of psychological safety and inclusion (Figure 5). We found that as the amount of verbal backchannels participants received increased, so did their psychological safety scores. Also, groups that had higher amounts of verbal backchanneling also had higher average ratings of psychological safety and inclusion. These results are the first, to our knowledge, to uncover significant relationships between team members' backchanneling behavior and their ratings of psychological safety and inclusion.

### 5.3. Robot Verbal Support Suppresses Human Verbal Backchannels to Outgroup Members

We then investigated what effects the verbal support from the robot had on the verbal backchanneling behavior of human team members. First, we found that, regardless of whether or not the group received verbal support from the robot, outgroup members received more verbal backchannels than ingroup members. This finding suggests that verbal backchannels may be expressed in greater volume to new group members as a way of making them feel welcome and included in the group. We also found an interaction between ingroup-outgroup membership and the presence of verbal support from the robot (Figure 6). When verbal support from the robot *was not present*, outgroup members received significantly more verbal backchannels from the other group members. However, when verbal support from the robot *was present*, we did not find any significant difference in the amount of verbal backchannels received by outgroup and ingroup members. This result surprised us, for we had expected that the verbal support from the robot would increase the verbal support, in the form of verbal backchanneling, that participants expressed toward the outgroup member. For example, in prior work, human group members that interacted with a robot that expressed vulnerability were also more likely to express vulnerability toward one another by explaining their mistakes and consoling one another (Strohkorb Sebo et al., 2018). Taking both of these results into consideration, what can explain how vulnerability from a robot encourages human team member vulnerability, while verbal support from a robot inhibits the verbal backchannels ingroup members gave to outgroup members?

One possible explanation for why verbal support from a robot suppresses the verbal backchannels an outgroup member receives is that there exists a ceiling effect of the amount of backchanneling that can reasonably exist in a conversation. It is possible that too many verbal backchannels might “block the airways” and interrupt or distract from the primary focus of the conversation. Therefore, in response to the added verbal support from the robot, human participants might reduce their verbal backchanneling. However, this idea is not supported by our results because it was only the verbal backchannels *toward the outgroup member* that were affected by the robot verbal support; there was no significant difference in the amount of verbal backchanneling between groups where the robot expressed verbal support and where the robot did not express verbal support.

Another possible explanation for why verbal support from a robot reduces the amount of verbal backchannels received by an outgroup member is that the robot backchanneling the outgroup member makes the robot an “outgroup” member as well. It is possible that the participants perceived the verbal support from the robot to the outgroup member as affiliation with the outgroup member, where the ingroup members would form one subgroup and the outgroup member and the robot would form the other subgroup. This possible formation of subgroups might further distance the outgroup member from the ingroup members, and therefore, make the ingroup members less likely to verbally backchannel the ingroup member. However, since the robot also showed verbal support for the ingroup members, and not the outgroup member exclusively, it is hard to conclude definitively that this is the case. In this experiment, the robot supported each participant equally: giving each participant approximately 6 targeted supportive utterances and verbally backchanneling each participant at the same fixed probability. Thus, it is difficult to conclude that the robot's verbal support of the outgroup member, that was no different than the robot's verbal support of each ingroup member, led the ingroup members to view the robot and the outgroup member as an outgroup, to whom they would backchannel less.

The most plausible explanation for why verbal support from a robot reduces the amount of verbal backchannels received by an outgroup member is that the ingroup members feel a *reduced obligation* to verbally backchannel the outgroup member in the presence of verbal support from the robot. When there is no verbal support from the robot, outgroup members receive significantly more backchannels than ingroup members. Thus, it seems likely that, without robot intervention, the ingroup members are making an effort to include their new group member (the outgroup member) and help them to feel comfortable within the group. When verbal support from the robot is present, it is possible that the ingroup members do not feel as large a responsibility to take actions to include the outgroup member because the robot is providing some of the verbal support to the outgroup member.

Regardless of the underlying cause of the reduction in verbal backchannels to the outgroup member in the robot verbal support conditions, it is unclear whether this reduction in backchannels toward the outgroup member had a positive, neutral, or negative effect on the interactions between team members. We observed no differences in the inclusion and psychological safety ratings between outgroup members who did and who did not receive robot verbal support. Therefore, we do not have a definitive answer with regards to whether the suppression of ingroup-to-outgroup verbal backchannels is positive or negative. It is possible that the robot is doing the ingroup members a favor with its verbal support by taking some of the burden of including and embracing the ideas of a new group member. However, it is also possible that when robot verbal support is present, the ingroup members become ‘lazy' and do not feel as great a need to affirm the contributions of the outgroup member, causing them to be less inclusive in the long term. We look forward to future work that can further investigate the effects of robot verbal support in human-robot teams, especially pertaining to ingroup-outgroup interactions.

### 5.4. Human Backchanneling and Robot Backchanneling

Beyond demonstrating the influence of robot verbal support on a human team, this work also provides further insight into the backchanneling behavior of both humans and robots in the context of collaborative teaming. Through our analysis of the correlations between human backchanneling and the team dynamics of psychological safety and inclusion (Figure 5), we discovered that (1) the total amount of backchannels an individual receives is positively correlated with their ratings of psychological safety, (2) the total amount of non-verbal backchannels an individual produces is negatively correlated with their ratings of psychological safety, (3) those who receive a high proportion of backchannels relative to their talking time report lower inclusion scores, and (4) groups that exhibit more verbal backchannels have higher average psychological safety and inclusion scores. These findings highlight the positive influence of verbal backchannels in collaborative interactions. For example, if you find yourself on a video call, it would likely benefit your team for you to un-mute and verbally backchannel your team members more often. Additionally, these results highlight human backchannels as a potentially powerful feature to predict team social dynamics.

Again with respect to human backchanneling, we found that outgroup members received more verbal backchannels than ingroup members in the experiment, regardless of whether or not the group received verbal support from the robot (Figure 6). This increase of backchannels to the outgroup member may either indicate that (A) people backchannel new group members more than older group members (in this experiment, the ingroup members had already interacted with one another before the arrival of the outgroup member), or (B) people backchannel outgroup members more than ingroup members. Either way, these results indicate that verbal backchannels likely correlate with the efforts of people to incorporate someone new or different into a group.

Our investigation into robot backchanneling also led to novel findings pertaining to the influence of verbal backchannels from a robot on a collaborative human team. Unlike human verbal backchannels, that exhibit significant positive correlations with psychological safety and inclusion, we did not observe the same with robot backchannels. Participants in groups receiving verbal support from the robot (targeted support, hints, verbal backchannels) had only marginally significantly higher psychological safety scores and no difference in inclusion scores than participants in groups without verbal support from the robot. It is possible that robot verbal backchannels did have a positive effect with respect to these team social dynamics, but perhaps not as strong of an effect as verbal backchannels from a human team member.

Additionally, we found that ingroup members verbally backchanneled the outgroup member less in the presence of robot verbal support. It is likely that the ingroup members felt less of a responsibility to be ‘good team members' by verbally backchanneling the outgroup member, since the robot was already doing so. This finding highlights a potentially negative outcome of robot verbal support and robot backchanneling: the suppression of verbal backchannels from ingroup to outgroup members. It is possible that if a robot were to verbally support the outgroup member even more than it did during this experiment, the ingroup members would feel even less responsibility to verbally backchannel the outgroup member, resulting in the outgroup member feeling less included and less psychologically safe. These results underscore the importance of understanding the effects of seemingly harmless and purely positive behavior from a robot, since, as we have shown, they may have negative consequences on subsequent human-to-human interactions within a group.

## 6. Conclusion

As robots increasingly join human teams, it is critical that we understand how their actions influence the social dynamics that are critical to team success (e.g., psychological safety, inclusion). In this work, we designed and ran a human-subjects experiment testing the efficacy of verbal support from a robot to positively shape psychological safety and inclusion within a human-robot team consisting of 2 human ingroup members, 1 human outgroup member, and 1 robot. Like prior work (Short and Matarić, 2017; Tennent et al., 2019; Traeger et al., 2020), we have demonstrated that a robot's actions can have a positive influence on human group members' interactions and dynamics: the targeted support from the robot encouraged more verbal contribution from outgroup members. Additionally, we have shown that verbal support from the robot inhibited the verbal backchannels that ingroup members directed toward outgroup members. This work raises the question of whether or not robot behavior should be encouraged when it suppresses the beneficial behavior that human members of the team express toward one another. With the growth of robots as members of human-robot teams, our results highlight the importance for future work to further understand how robot actions may influence human-robot team interactions, especially over longer periods of time.

## Data Availability Statement

The raw data supporting the conclusions of this article will be made available by the authors, without undue reservation.

## Ethics Statement

The studies involving human participants were reviewed and approved by Yale University Internal Review Board. Written informed consent to participate in this study was provided by the participant and also, if the participant was under the age of 18, the participants' legal guardian/next of kin. Additionally, written informed consent was obtained from the participant and also, if the participant was under the age of 18, the minor(s)' legal guardian/next of kin for the publication of any potentially identifiable images or data included in this article.

## Author Contributions

The idea of a robot being used to improve the social dynamics of a group was developed by SS and BS. The human subjects experiment was designed by SS, BS, LD, and NC. SS, LD, NC, and MS programmed the robot and designed the survival task under the guidance of BS. SS recruited and ran the participants through the experimental protocol. SS and ML annotated the participant videos for instances of backchanneling. SS performed the statistical analysis of the data. The manuscript was drafted by SS and was reviewed and edited by BS, LD, NC, ML, and MS. All authors contributed to the article and approved the submitted version.

## Conflict of Interest

The authors declare that the research was conducted in the absence of any commercial or financial relationships that could be construed as a potential conflict of interest.

## Footnotes

^1^A portion of the results presented in th is paper were previously published as: Strohkorb Sebo, S., Dong, L. L., Chang, N., and Scassellati, B. (2020). “Strategies for the inclusion of human members within human-robot teams,” in *Proceedings of the 2020 ACM/IEEE International Conference on Human-Robot Interaction, HRI'20* (New York, NY: Association for Computing Machinery), 309–317. (Strohkorb Sebo et al., 2020)
